# A retrospective study of focal segmental glomerulosclerosis: clinical criteria can identify patients at high risk for recurrent disease after first renal transplantation

**DOI:** 10.1186/1471-2369-14-47

**Published:** 2013-02-22

**Authors:** Rutger JH Maas, Jeroen KJ Deegens, Jan AJG van den Brand, Elisabeth AM Cornelissen, Jack FM Wetzels

**Affiliations:** 1 Department of Nephrology 464, Radboud University Nijmegen Medical Center, PO Box 9101, Nijmegen, HB 6500, The Netherlands; 2 Department of Pediatric Nephrology, Radboud University Nijmegen Medical Center, Nijmegen, The Netherlands

**Keywords:** FSGS, Recurrence, Renal transplantation, Risk factors

## Abstract

**Background:**

Focal segmental glomerulosclerosis (FSGS) is a frequent cause of end-stage renal disease. Renal transplantation in patients with FSGS is often complicated by disease recurrence, which is associated with poor outcome. There are no tests that reliably predict recurrence of FSGS after transplantation. The aim of this study was to evaluate if clinical criteria can identify patients at high risk for recurrent disease.

**Methods:**

We retrospectively studied 94 patients who received a first renal transplant at a median age of 37 years (range 5–69 years). Patients were assigned to one of three groups: familial or genetic FSGS (group I; n=18), secondary FSGS (group II; n=10) and idiopathic FSGS (group III; n=66). Pretransplant clinical characteristics were analyzed to determine predictors of a recurrence after transplantation.

**Results:**

FSGS only recurred in patients with idiopathic FSGS (group III; 42%). Patients with a recurrence had a significantly lower serum albumin, higher 24-hour proteinuria and higher estimated glomerular filtration rate at diagnosis. Serum albumin at diagnosis was the only independent predictor of a recurrence in patients with idiopathic FSGS. Patients with recurrent FSGS had more acute rejection episodes (54% vs. 27%, *P* =0.02) and lower five year graft survival compared to patients without a recurrence (50 vs. 82%, *P* <0.01).

**Conclusions:**

Clinical criteria allow identification of patients at high risk of recurrent FSGS after renal transplantation. This information can be used in the counseling and management of patients with FSGS.

## Background

Focal segmental glomerulosclerosis (FSGS) is a histological diagnosis and not a single disease entity. FSGS can occur at any age and presenting features include a variable degree of proteinuria, microscopic hematuria, hypertension and decreased glomerular filtration rate. While numerous causes for FSGS have been identified, still most patients are diagnosed with idiopathic FSGS [1,2]. Progression to end-stage renal disease is common, especially in patients with decreased glomerular filtration rate or nephrotic range proteinuria at presentation [1]. In case of end-stage renal disease, renal transplantation is usually the treatment of choice. After renal transplantation, FSGS is reported to recur in 15-52% of first grafts [3-10]. Significant proteinuria often progressing to nephrotic range proteinuria in the first days after transplantation heralds a recurrence in virtually all patients [11]. Graft biopsy may either disclose normal glomeruli or typical FSGS lesions on light microscopy, and effacement of the epithelial foot processes on electron microscopy [12,13]. Without treatment, recurrent FSGS leads to early graft failure in more than 50% of patients [14,15]. There is strong evidence that a circulating permeability factor is responsible for glomerular injury in many patients with recurrent FSGS after transplantation [16,17]. The suggestion of a circulating permeability factor has led to the use of plasma exchange for the treatment of recurrent FSGS. When initiated early after onset of proteinuria, plasma exchange can induce a remission of proteinuria and portends a better prognosis [18,19]. Therefore, accurate identification of patients at risk for recurrent FSGS is an important issue.

We report a retrospective analysis of all patients with FSGS, who received a first renal transplant in our center. The purpose of the study was to define clinical criteria which would allow to identify patients at high risk for recurrent disease during pretransplant counseling.

## Methods

We identified all patients with FSGS who received a first renal transplant in our center in the period 1968–2009. Patients were included if the diagnosis of FSGS was confirmed on a native renal biopsy or if they had otherwise unexplained proteinuria and a first degree relative with biopsy proven FSGS. Patients with FSGS lesions secondary to other glomerular diseases including IgA nephropathy, membranous nephropathy and Alport disease were excluded.

For the purpose of the study, the medical records and pathology reports were reviewed. The Hospital Research Ethics Committee deemed formal committee review of the study unnecessary. Data collection was performed according to the Dutch Code of Conduct for Health Research.

Patients were assigned to one of three groups: Group I: patients with a first degree relative with FSGS and/or identified gene mutations that cause FSGS. Group II: patients with secondary FSGS based on previously published criteria (with exclusion of cases assigned to group I) [1]. Group III: patients without evidence of genetic or secondary FSGS. This group is referred to as idiopathic FSGS. Assignment to a group was done without knowledge of transplantation outcome.

### Definitions

Date of onset of FSGS in native kidneys was defined as date of first presentation. Date of diagnosis was recorded as biopsy date. Glomerular filtration rate was estimated with the abbreviated MDRD study equation in adults and the Schwartz formula in children (age < 16 years) [20,21]. Definite recurrence of FSGS was defined as the occurrence of progressive proteinuria after transplantation, with 1) evidence of FSGS on light microscopy or 2) normal appearing glomeruli on light microscopy, but foot process effacement on electron microscopy. A probable recurrence was defined as proteinuria with normal glomeruli on light microscopy and no electron microscopy performed. Acute antibody mediated rejection has been described as a possible cause of early heavy posttransplant proteinuria [22]. Any histologic signs of antibody mediated rejection were therefore recorded. Delayed graft function was defined as need for dialysis within the first week after transplantation.

### Statistical analysis

Values were expressed as median with range or mean ± SD. Statistical differences between groups were assessed with Chi-square or Fisher exact test, unpaired Student’s *t* test or Mann Whitney U test, where appropriate. We used a binary probit model to identify independent predictors of recurrent FSGS [23]. To improve the validity of multivariate data analyses, 100 data sets were created with imputed values for albumin, 24-hour proteinuria and estimated glomerular filtration rate (eGFR) using Stata’s ice procedure [24]. Ordinary least squares regression analysis was used to impute missing values. This model included age, gender, eGFR, proteinuria, serum albumin, and recurrence after transplantation [25]. The association between predictor and imputed variables were assumed to be linear, no interactions were included as effect modification was deemed unlikely to be present in the final analysis. Kaplan–Meier curves and log-rank tests were used for description and comparison of graft survival. A two-sided *P*-value <0.05 was considered as the level of statistical significance for all tests. The analyses were performed with the use of SPSS 16.0 for Windows (SPSS inc, Chicago, IL, USA) and Stata 10 for Windows (Stata Corporation, Texas, USA).

## Results

### Baseline characteristics

We identified 94 patients with FSGS who received a first renal transplant. In 92 patients, the diagnosis was proven by renal biopsy. In addition, in two patients the diagnosis FSGS was based on the presence of proteinuria and the family history of a first degree relative with biopsy proven FSGS. Baseline characteristics are shown in Table 1. Eighty-five patients were native Dutch. The remaining patients were Turkish (n=3), Moroccan (n=1), Dutch Antillian (n=1), Sri Lankan (n=1), Indonesian (n=1), Iranian (n=1) and Somalian (n=1). Thirty-two patients were younger than 16 years at onset of FSGS. Eighteen patients were diagnosed with familial/genetic FSGS (group I). Mutations were identified in four of them. One patient had a three-allelic mutation (compound heterozygous mutations in the *NPHS2*-gene and one mutation in the *NPHS1*-gene), two related patients had homozygous mutations in the *NPHS2*-gene and one patient had a mutation in the *WT1*-gene. A secondary cause was identified in 10 patients (group II): renal agenesis (n=3), traumatic kidney injury (n=1), hypoplasia of one kidney (n=1), reflux nephropathy (n=2), chronic pyelonephritis (n=1), a history of intravenous heroin abuse (n=1), and Hajdu-Cheney syndrome (n=1) [26]. Idiopathic FSGS was diagnosed in 66 patients (group III).

**Table 1:** Baseline characteristics of patients with FSGS and comparison of baseline characteristics between patients with and without FSGS recurrence

		**Total population (n=94)**	**Recurrence (n=28)**	**No recurrence (n=66)**	***P*-value (difference recurrence vs no recurrence group)**
Sex (M/F)		51/43	16/12	35/31	0.82
Age at onset (yr)		26 (1–61)	19 (4–57)	26 (1–61)	0.63
Age at diagnosis (yr)^*^		28 (2–62)	23 (5–57)	29 (2–62)	0.25
Serum albumin at diagnosis (g/l)		27 ± 11 (n=60)	20 ± 9 (n=20)	31 ± 10 (n= 40)	**<0.01**
24-hour protein exretion (g)		8.0 ± 7.3 (n=42)	11.7 ± 8.8 (n=15)	5.9 ± 5.5 (n=27)	**0.01**
eGFR at diagnosis^†^ (ml/min/1.73 m^2^)		81 ± 53 (n=63)	104 ± 50 (n=21)	69 ± 51 (n=42)	**0.01**
Cause of FSGS	*Familial/geneticFSGS (group I)*	18 (19%)	0	18	**<0.01**
	*Secondary FSGS (group II)*	10 (11%)	0	10	
	*Idiopathic FSGS (group III)*	66 (70%)	28	38	
Bilateral nephrectomy before transplantation		6	1	5	0.67
Interval onset to diagnosis^*^ (yr)		0.5 (0–31)	0.3 (0–6.9)	0.8 (0–31.0)	0.07
Interval diagnosis to ESRD (yr)		3.9 (0.1-32.4)	4.9 (0.1-16.8)	3.7 (0.1-32.4)	0.96
Interval ESRD to transplantation		1.4 (0–9.3)	1.2 (0–4.7)	1.4 (0–9.3)	0.53
Age at transplantation (yr)		37 (5–69)	32 (10–62)	38 (5–69)	0.17
Donor Source	Deceased	75	24	51	0.57
	Living unrelated	7	1	6	
	Living related	12	3	9	
Donor age (yr)		35.5 (0–68)	36.5 (5–64)	35.5 (0–68)	0.91
Number of HLA mismatches		2.0 ± 1.2	2.0 ± 1.2	2.0 ± 1.2	0.77
Initial immunosuppressive regimen	P + CsA	54	18	36	0.38
	P + Aza	11	5	6	
	P + CsA + MMF	13	2	11	
	P + tacro + MMF	14	3	11	
	Dacl + tacro + MMF	2	0	2	

Data are given as n(%), means ± SD or median (range).

*age at diagnosis is based on biopsy date. Two patients with familial FSGS were not biopsied. For these patients, age at diagnosis equals age of first presentation.

†eGFR was calculated with the abbreviated MDRD study equation in adults and with Schwartz’ formula in children.

P = prednisone, CsA = cyclosporine, Aza = azathioprine, MMF = mycophenolate mofetil, Tacro=tacrolimus, Dacl = daclizumab.

eGFR = estimated glomerular filtration rate, ESRD = end-stage renal disease.

A diagnosis of FSGS was made at a median of 6.0 years (0.3-36.9 years) before renal transplantation. Patients were on dialysis for a median of 1.4 years (0–9.3 years) before transplantation. Bilateral nephrectomy was performed before transplantation in six patients, all were children. Four patients underwent preemptive renal transplantation.

### Recurrence after renal transplantation

FSGS recurred in 28 patients (30%; Table 1). Twenty-four patients had a definite recurrence of FSGS and four patients had a probable recurrence. Typical FSGS lesions were seen in light microscopy in biopsies of 11 patients. In none of the patients there was evidence of acute transplant glomerulopathy. Peritubular capillary C4d staining was performed in biopsies of six patients with a recurrence, and all were negative. Three probable recurrences occurred within the first month after transplantation, and all had nephrotic range proteinuria. All recurrences occurred in patients with idiopathic FSGS (group III; *P* <0.01). Median amount of proteinuria at the time of graft biopsy in patients with a recurrence was 5.0 g/day (interquartile range 3.6 - 6.9 g/day). The median time to recurrence was 6 days (0–2082 days) after transplantation. In two patients the diagnosis of recurrence was made more than six months after renal transplantation. The first patient, a 32-year old woman, had a probable recurrence more than five years after transplantation after cyclosporine monotherapy was replaced by azathioprine and prednisone because of cyclosporine toxicity. Shortly thereafter massive proteinuria occurred. Initiation of plasma exchange led to a remission of proteinuria, however prolonged plasma exchange was necessary to sustain the remission of proteinuria. The other late recurrence was a definite recurrence which occurred four years after transplantation in an 18 year-old patient. No inciting event was found. He was treated with ACE-inhibitors and had only slight proteinuria and a reasonable graft function after a follow-up of 17 years.

Compared to patients without a recurrence, delayed graft function occurred more often in patients with recurrent FSGS, although this difference did not reach statistical significance (25% vs. 12%, *P* =0.13). Patients with recurrent FSGS had significantly more biopsy-proven acute rejection episodes (54% vs. 27%, *P* =0.02). There were no significant differences in HLA-mismatches and immunosuppressive regimens. Graft survival at five years was significantly lower in patients with recurrent FSGS compared to patients without a recurrence (50% vs. 82%; *P* <0.01). Plasma exchange for the treatment of recurrent FSGS was introduced in 1994. We have previously shown an improved outcome in patients treated with plasma exchange compared to historical controls [18]. In the current study nine out of 28 patients with recurrences were treated with plasma exchange.

### Clinical prediction of recurrent FSGS

To determine clinical factors associated with FSGS recurrence, we compared baseline characteristics of patients in group III with and without a recurrence of FSGS (Table 2). Patients with familial or secondary FSGS were excluded because a recurrence did not occur in these patient groups. Patients with a recurrence had a significantly lower serum albumin, higher 24-hour proteinuria and higher eGFR at diagnosis (Table 2). They also had a shorter interval between presentation and renal biopsy than patients without a recurrence. The interval between first renal biopsy and end-stage renal disease did not differ significantly between patients with and without a recurrence, nor did age at diagnosis and donor age.

**Table 2:** Demographic and transplant-related factors, and laboratory results at diagnosis in patients with idiopathic FSGS (group III) with and without a recurrence

		**Recurrence**	**No recurrence**	***P*-value**
N		28	38	
Sex (M/F)		16/12	24/14	0.80
Age at presentation (yr)		19 (4–57)	28 (1–61)	0.45
Age at diagnosis (yr)		23 (5–57)	34 (2–61)	0.13
Body mass index at diagnosis in patients >16 yr (n=48)		24.6 ± 3.6 (n=12)	25.0 ± 3.5 (n=26)	0.76
eGFR at diagnosis (ml/min/1.73 m^2^)		104 ± 50 (n=21)	53 ± 22 (n=22)	**<0.01**
Serum albumin at diagnosis (g/l)		20 ± 9 (n=20)	33 ± 10 (n=24)	**<0.01**
24-hour protein excretion (g)		11.7 ± 8.8 (n=15)	4.6 ± 2.7 (n=14)	**<0.01**
Interval onset to diagnosis (yr)		0.3 (0–6.9)	1.0 (0–22)	**<0.01**
Interval diagnosis to ESRD (yr)		4.9 (0.1-16.8)	3.7 (0.2-32.4)	0.96
Interval ESRD to Tx (yr)		1.2 (0–4.7)	1.5 (0–9.3)	0.34
Age at transplantation (yr)		32 (10–62)	44 (5–68)	0.06
Donor age (yr)		36.5 (5–64)	38 (0–68)	0.60
Number of HLA mismatches		2.0 ± 1.2	2.1 ± 1.2	0.81
Donor source (n)	Deceased	24	27	0.35
	Living unrelated	1	4	
	Living related	3	7	
Baseline immunosuppressive regimen (n)	P, CsA	18	23	0.64
	P, Aza	5	4	
	P, CsA, MMF	2	2	
	P, tacro, MMF	3	8	
	Dacl, tacro, MMF	0	1	

Data are given as n(%), means ±SD or median (range).

P = prednisone, CsA = cyclosporine, Aza = azathioprine, MMF = mycophenolate mofetil, Tacro=tacrolimus.

Dacl = daclizumab, eGFR = estimated glomerular filtration rate, ESRD = end-stage renal disease, Tx = transplantation.

Serum albumin, eGFR and 24-hour proteinuria were analyzed as predictors in the binary probit model. Because there was significant multicollinearity between albumin and 24-hour proteinuria, these parameters were analyzed separately with eGFR. In the complete case analysis of eGFR and serum albumin (n=39), serum albumin was an independent predictor with a relative risk of 0.93 per incremental g/l (95% confidence interval 0.88-0.98). In the complete case analysis of eGFR and 24-hour proteinuria (n=26), 24-hour proteinuria was a significant predictor with a relative risk of 1.36 per incremental g/24 hours (95% confidence interval 1.05-1.77). eGFR was a significant but weak predictor in the complete case analysis. After imputation serum albumin was the only independent predictor of a recurrence with a relative risk 0.92 per incremental g/l (95% confidence interval 0.88-0.96).

**Figure 1:**
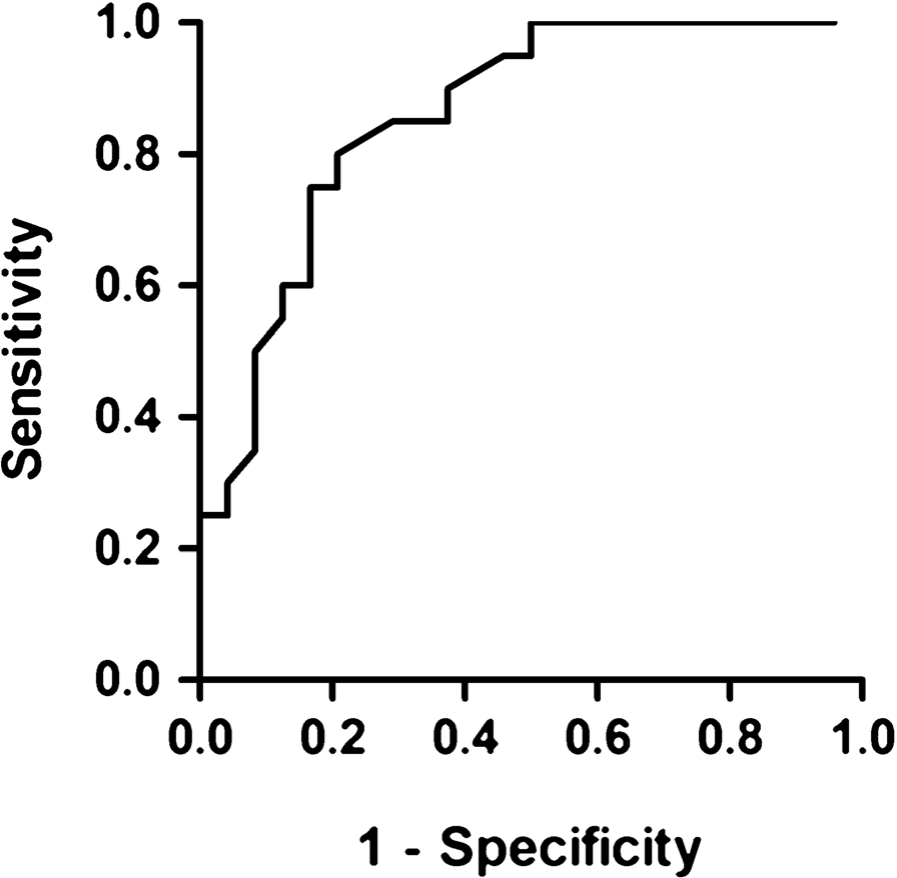
**ROC curve of the predictive value of serum albumin at diagnosis for FSGS recurrence AUC 0.87 (0.75-0.97); *P*<0.0001.**

The percentage of recurrences by serum albumin level at diagnosis in the idiopathic FSGS group is shown in Table 3. The ROC curve for the predictive value of serum albumin at diagnosis for FSGS recurrence is shown in Figure 1.

**Table 3:** Recurrence rates in patients with idiopathic FSGS according to serum albumin concentration at diagnosis

**Serum albumin (g/l)**	**Number of cases**	**Patients with recurrences (%)**
>35	12	0
25-35	14	6 (43%)
<25	18	14 (78%)
Missing	22	8 (36%)

## Discussion

Many factors have been associated with recurrence of FSGS after renal transplantation, including a young age at diagnosis, a rapid progression to end-stage renal disease, loss of a previous renal graft from recurrent disease, and prior bilateral nephrectomy [3,4,6,15,27]. Except for recurrence in a previous graft none of these factors is highly predictive in an individual patient [28]. Since recurrent FSGS has been associated with a circulating plasma factor, in vitro determination of plasma factor(s) that induce permeability to albumin in isolated glomeruli has also been used to predict recurrence [6,16]. Although initial reports showed good correlations between in vitro tests and recurrent disease in renal grafts, later studies reported less robust correlations and less specificity [29]. Recently, Wei *et al.* have described serum soluble urokinase receptor (suPAR) as a circulating factor that may cause FSGS [17]. They reported that higher concentrations of suPAR before transplantation are associated with an increased risk for recurrence after transplantation. However, there was a large degree of overlap between pretransplant suPAR in patients with recurrent and nonrecurrent FSGS. We have recently shown that suPAR is not a specific marker for idiopathic FSGS [30]. In the absence of a reliable diagnostic test that can predict FSGS recurrence after transplantation, we evaluated whether clinical criteria can improve pretransplant risk assessment.

In our study FSGS recurred only in patients assigned to group III (idiopathic FSGS). In this group recurrence rate was 42%. The lower recurrence rate reported in many studies may be related to inclusion of patients with secondary/genetic FSGS [3-5,9]. Except for patients with congenital nephrotic syndrome (*NPSH1*) in which anti-nephrin antibodies may be involved, FSGS rarely recurs in patients with genetic FSGS [31,32]. With the identification of mutations in podocyte-associated genes it has become apparent that an increasing number of patients originally thought to have idiopathic FSGS, actually have a form of genetic FSGS [2]. In children with FSGS, genetic testing strategy in our center has involved sequential testing for all mutations known at the time. Most children in this cohort received a transplant before any FSGS associated mutations were identified. In adult-onset sporadic FSGS, most of the currently known FSGS associated mutations are rare [33]. Still, we cannot exclude the possibility that some patients assigned to our group III in fact had an unidentified genetic mutation.

In the early phase of recurrent FSGS after transplantation, glomeruli often appear normal on light microscopy, but display diffuse foot process effacement on electron microscopy. We used a definition of a probable recurrence if glomeruli appeared normal but electron microscopy was not performed. Nevertheless, recurrent FSGS was in fact the most likely diagnosis in these cases, as all patients had nephrotic range proteinuria. Moreover, previous studies have used similar definitions [13]. Although acute antibody mediated rejection can cause postttansplant proteinuria, we consider this an unlikely event. There was no evidence of acute transplant glomerulopathy in light microscopy, and in available biopsies C4d stainings were negative.

Risk factor analysis using a binary probit model identified serum albumin at diagnosis as an independent predictor of recurrence. In patients with idiopathic FSGS (group III) and a serum albumin <25 g/l at diagnosis recurrence rate was high (78%), whereas FSGS did not recur in patients with a normal serum albumin (>35 g/l) at diagnosis. This finding supports the present notion among many clinicians that a more severe nephrotic syndrome in patients with FSGS is associated with a higher recurrence risk. To our knowledge, this is the first study to demonstrate the predictive value of serum albumin at diagnosis in recurrent FSGS. This is likely due to the fact that previous studies have never reported pretransplant serum albumin.

Admittedly, serum albumin was not available in 22 patients with idiopathic FSGS which may have biased results. To account for these missing values, we used imputed values in the binary probit model. It has been shown that a properly performed multiple imputation gives less biased results compared to traditional complete case analysis [34]. Recurrence after transplantation was used as a predictor variable because multiple imputation with the outcome has been shown to yield more valid results [25].

Previously, Praga et al. showed that a normal serum albumin was suggestive of FSGS secondary to hyperfiltration in patients with FSGS in their native kidneys and nephrotic range proteinuria. Hyperfiltration in these patients was due to severe obesity (BMI ≥ 35 kg/m^2^), vesicoureteral reflux, or renal mass reduction. In our study, none of the children were obese, and none of the adult patients in the idiopathic FSGS group had a body mass index over 35 kg/m^2^. Moreover, patients with reflux or renal mass reduction were assigned to group II. Nevertheless, it is possible that at least some of the patients in group III, specifically those with a normal serum albumin and nephrotic range proteinuria at diagnosis, had glomerular hyperfiltration that would not be expected to recur in the renal transplant. Because we were unable to identify an underlying secondary cause with certainty, we assigned these patients to group III (idiopathic FSGS). In daily clinical practice these patients would also be diagnosed with idiopathic FSGS.

In the past, living related donor transplantations have often been avoided in patients with FSGS, because of fear that recurrent FSGS in the renal allograft would lead to premature graft loss. However, this fear has become less justified since therapeutic plasma exchange has been shown to improve outcome in patients with recurrent FSGS. Moreover, data from the USRDS study revealed that living related donor transplantation has no association with graft loss from recurrent FSGS [35]. Rather, living related donor transplant is associated with a graft survival advantage for living donor kidneys over deceased donor kidneys. Furthermore, as confirmed by our study, the rate of recurrence among recipients of living or deceased donor kidneys is similar [5,9,14,36-38].

The immunosuppressive regimen did not influence the risk of recurrent disease in our study. In particular, the use of cyclosporine was not associated with a decreased recurrence rate. In children, there is limited evidence that recurrent FSGS can be successfully treated with high-dose intravenous or oral cyclosporin [39,40]. However, cyclosporine does not appear to prevent recurrence in the transplant when given as part of the initial immunosuppressive regimen [38,41,42].

A rapid clinical course to end-stage renal disease, a previously reported risk factor for recurrence, did not influence the recurrence rate in our study [4,14,15,37]. Several other studies also observed no differences between the rapidity of progression of the initial disease and recurrent disease [5,7-9,38]. Although speculative, differences in pre-transplant immunosuppressive therapy may help explain the discrepancy between the above mentioned studies. Pardon *et al.* and Hickson *et al.* showed that recurrent disease occurred more often in patients treated with cyclosporine before transplantation, whereas time to end stage renal disease did not influence recurrence rate [7,8]. Although these patients eventually developed end-stage renal disease, cyclosporine may have slowed progression by reducing proteinuria. As recently shown, cyclosporine can reduce proteinuria due to a direct effect on podocyte function, which appears to be related to a stabilization of the actin cytoskeleton in podocytes [43]. In contrast, these patients may progress rapidly to end-stage renal disease if not treated with cyclosporine.

Several investigators have identified bilateral nephrectomy before transplantation as a risk factor for recurrence [4,27]. This may reflect a more aggressive disease with a higher propensity to recur. In our study, we were not able to confirm this finding because bilateral nephrectomy was only performed in six patients. We only advise nephrectomy in patients with end-stage renal disease and severe, persistent nephrotic syndrome refractory to medical treatment.

Recurrent FSGS was associated with decreased graft survival: within five years, half of the patients with recurrent FSGS lost their renal graft compared to only 18% of patients without recurrent FSGS. It is important to note that most patients included in our cohort were transplanted in the era before plasma exchange was applied as treatment of FSGS recurrence. We and others have shown that sustained remissions resulting in better renal survival can be attained after treatment with plasma exchange [18,19,41]. The higher incidence of acute rejection episodes in patients with FSGS recurrence may also have contributed to their decreased graft survival. In addition to acute rejections, delayed graft function also appeared to occur more often in patients with recurrent FSGS, although this difference was not statistically significant. It is not clear how FSGS recurrence, delayed graft function, and acute rejection are intertwined. Kim et al. reported that both recurrence of FSGS and delayed graft function were independently associated with an increased incidence of acute rejection in patients with end-stage renal disease due to FSGS [44]. Furthermore, there was an increased rate of early graft dysfunction in their FSGS patients, which was assumed to be a consequence of immediate recurrence. In our study, the increased number of acute rejection episodes may be due to a higher surveillance rate. Patients with an FSGS recurrence had a mean of 1.5 renal graft biopsies per patient in the first year after transplantation, whereas the patients without a recurrence had only 0.5 biopsies.

## Conclusions

Clinical criteria can identify a group of patients at high risk for recurrent FSGS after renal transplantation. Serum albumin at diagnosis predicts recurrence rate with a 78% risk of recurrence in patients with serum albumin concentration <25 g/l. Admittedly our study is limited by the retrospective design and missing data. Prospective studies are needed to confirm our findings. Nevertheless, our data can already be used to provide better counseling of patients with FSGS before renal transplantation. High risk patients should be closely monitored for proteinuria, especially in the first weeks after transplantation, to enable early initiation of plasma exchange.

## Competing interests

The authors declare that they have no competing interests.

## Authors’ contributions

RJHM conceived and designed study, collected data, analyzed data, and wrote the manuscript. JKJD: conceived and designed study, analyzed data, and participated in writing the manuscript. JAJGB: participated in the design of the study, and performed statistical analyses in Stata. EAMC: collected data, and participated in writing the manuscript. JFMW: participated in design of the study, and participated in writing the manuscript. All authors read and approved the final manuscript.

## Pre-publication history

The pre-publication history for this paper can be accessed here:

http://www.biomedcentral.com/1471-2369/14/47/prepub
